# The Effect of Radixin on the Function and Expression of Organic Anion Transporting Polypeptide 1B1

**DOI:** 10.3390/biology14070744

**Published:** 2025-06-23

**Authors:** Chunxu Ni, Longxia Tang, Xuyang Wang, Zichong Li, Mei Hong

**Affiliations:** 1 College of Life Sciences, South China Agricultural University, Guangzhou 510642, China; 2 Guangdong Provincial Key Laboratory for the Development Biology and Environmental Adaptation of Agricultural Organisms, South China Agricultural University, Guangzhou 510642, China

**Keywords:** OATP1B1, radixin, oligomerization, protein kinase C, transport function

## Abstract

Organic anion transporting polypeptide 1B1 (OATP1B1) is selectively expressed at the basolateral membrane of human hepatocytes and plays a crucial role in the absorption of various xenobiotic compounds, including many important clinical drugs. Oligomerization with regulatory proteins is a common mechanism for regulating membrane protein functions. In the present study, we found that knocking down the scaffold protein radixin, which is the major member of the ERM family expressed in the liver, significantly enhanced the uptake function of OATP1B1. On the other hand, the overexpression of the phospho-mimic form of radixin (radixin-D) reduced the uptake function and cell surface level of OATP1B1, while the wild-type and phospho-dormant form of radixin (radixin-A) did not exhibit the same effect. Further investigation revealed that radixin interacts with OATP1B1. Activation of protein kinase C (PKC), which our previous study showed accelerates the internalization of OATP1B1, was found to increase the phosphorylation level of radixin associated with OATP1B1. The knockdown of radixin significantly diminished the suppressive effect of PKC on the function and cell surface levels of OATP1B1. These results suggested that OATP1B1 forms complexes with radixin, which may be phosphorylated by PKC, leading to reduced cell surface expression and activity of the transporter.

## Simple Summary

Organic anion transporting polypeptide 1B1 (OATP1B1) is a key hepatic uptake transporter for drug uptake. Change of OATP1B1 function will affect the pharmacokinetics of various clinically important therapeutic agents. The present study reveals that the scaffold protein radixin regulates the function OATP1B1. Knockdown of radixin significantly increased OATP1B1-mediated substrate uptake. Conversely, overexpression of a phospho-mimic radixin mutant (radixin-D) suppressed both transport activity and cell surface expression of OATP1B1, while wild-type or phospho-dormant (radixin-A) forms had no effect. Importantly, radixin directly interacted with OATP1B1, and protein kinase C (PKC) activation enhanced the phosphorylation of radixin bound to the transporter. Radixin knockdown abolished PKC-induced suppression of OATP1B1 function and surface levels, demonstrating that PKC-mediated radixin phosphorylation is essential for this regulatory mechanism. These findings establish radixin as a dynamic modulator of OATP1B1, the phosphorylation of which influences the location and activity of the transporter.

## 1. Introduction

Organic anion transporting polypeptide 1B1 (OATP1B1) is the major organic anion transporting polypeptide family member that is specifically expressed at the basolateral membrane of human hepatocytes. OATP1B1 is well-recognized as a key determinant in the absorption, distribution, and excretion of many clinically important drugs [1]. The three-dimensional structure of OATP1B1 has been resolved by cryo-electron microscopy (cryo-EM) recently, which facilitates a more comprehensive understanding of the structure–function relationship of the transporter [2,3]. Although the substrates of OATP1B1 and its involvement in drug–drug interactions (DDIs) have been extensively investigated, information regarding the cellular regulation of OATP1B1 is quite limited [4].

Oligomerization is a common regulatory regime for the regulation of membrane protein functions [5]. Quite a few transporter proteins have been demonstrated to exist as oligomers, either as homo-oligomers or hetero-oligomers. A previous study in our laboratory found that OATP1B1 forms homo-oligomers in intact cells. The oligomerization status may affect OATP1B1 transport function at the estrone sulfate high-affinity binding site, while at the low-affinity component of the substrate monomers of the multimeric complexes may function independently [6]. OATP1B3, another OATP family member that is specifically expressed in human hepatocytes, was demonstrated to form homo-oligomers as well. In addition, it was shown that OATP1B3 may hetero-oligomerize with other hepatic transporters such as OATP1B1 or Na^+^/taurocholate co-transporting polypeptide (NTCP), suggesting a potential co-regulation of the involved transporters [7]. Transporters were also found to be associated with regulatory proteins and form hetero-oligomers. Wang et al. identified PDZK1 as the major interacting protein of rat Oatp1a1. Disruption of such an association can affect the proper targeting and sorting of Oatp1a1 to the basolateral membrane, which in turn affects the proper function of the transporter [8]. It was demonstrated that both PDZK1 and NHERF1 directly interact with OATP1A2 and regulate its function by modulating the internalization and stability of the protein [9]. Unlike OATPs that contain a Class I PDZ binding domain at their carboxyl termini [10], OATP1B1 and 1B3 do not contain such a PDZ binding domain at their C-termini. However, a recent study demonstrated that the C-terminal ETHC sequence of OATP1B1 plays a critical role in its interaction with PDZK1, which in turn regulates plasma membrane targeting of the transporter [11].

The ERM proteins are an evolutionarily conserved group of three related proteins (ezrin, radixin, and moesin) that interact with the plasma membrane through a common FERM (Four-point-one, ezrin, radixin, moesin) domain. ERMs have been shown to regulate cellular processes including the reorganization of actin cytoskeleton, cell survival, membrane dynamics, cell migration, adhesion, and regulation of membrane protrusion [12]. It was demonstrated that multidrug resistance protein 2 (Mrp2) is decreased in the bile canalicular membranes of *Rad^−/−^* mice. Further analysis revealed that radixin may associate with the cytoplasmic C-terminal domain of Mrp2, and the interaction of radixin with C-Mrp2 is required for the transporter to localize correctly to the apical membranes in the liver, at which the secretion of conjugated bilirubin occurs [13]. Mrp2 was also found to interact with ezrin, the phosphorylation status of which affected the association of Mrp2 and ezrin, and in turn decreased the cell surface expression of the transporter [14].

Among the three ERMs, radixin is the predominant form found in the liver and has been shown to affect the function of membrane proteins such as P-glycoprotein [15] and Mrp2 [13]. In the present study, it was found that the knockdown of radixin increased the uptake function of OATP1B1. Further investigation revealed that radixin directly interacts with the transporter, and that the phosphorylation of radixin reduced the cell surface expression of OATP1B1. In addition, protein kinase C (PKC), which was previously shown to play a regulatory role for OATP1B1 [16], is likely involved in the phosphorylation of radixin.

## 2. Materials and Methods

### 2.1. Materials

Biotinylation reagents sulfosuccinimidyl 2-(biotinamido)-ethyl-1, 3-dithiopropionate (NHS-SS-biotin), streptavidin agarose beads, cell culture medium, and enzymes for molecular biology were purchased from ThermoFisher Scientific Inc. (Waltham, MA, USA). Antibodies for the detection of GFP (#AF0159), radixin (#AF1984), and flag (#AF0036) were purchased from Beyotime Biotech Inc. (Shanghai, China). The phospho-ezrin (Thr567)/radixin (Thr564)/moesin (Thr558) (48G2) (#3726) antibody was purchased from Cell Signaling Technology (Danvers, MA, USA). All other chemical reagents were obtained from Sigma-Aldrich (St. Louis, MO, USA) except otherwise stated.

### 2.2. Cell Culture and Transfection of Plasmid Constructs into Cells

HepG2 cells and HEK293 cells were cultured at 37 °C in a 5% CO_2_ environment using Dulbecco’s Modified Eagle’s Medium, supplemented with 10% fetal bovine serum. The cells were seeded in different plates and allowed to sit overnight before transfection. Transfection was performed using a LipofectAMINE 2000 reagent (Thermo Fisher, Waltham, MA, USA). After transfection, the cells were incubated for 48 h at 37 °C before proceeding with further analyses.

### 2.3. Generation of Knockdown Cell Lines

Short hairpin RNAs (shRNAs) that target the in-frame sequences ATGAGCATGA CGACAAGTTAA (shRadixin-1) and GCCTTATGTATGGGAAACCAT (shRadixin-2) of radixin were designed. The shRNAs were cloned into the lentiviral vector pLKO.1 and applied for cell transfection. The cells that had the shRNA vector incorporated were selected with puromycin (1 µg/mL). The protein level of radixin was checked before the knockdown cells were utilized for further experiments.

### 2.4. Uptake Function Measurement, Cell Surface Biotinylation, and Western Blotting

The uptake function and cell surface expression of OATP1B1 were analyzed as previously described [17]. In brief, cells were incubated in a 48-well plate at 37 °C with an uptake solution containing 2′, 7′-dichlorofluorescein (DCF) for 5 min (or 2 min for kinetic analysis). The uptake was terminated by adding ice-cold phosphate-buffered saline (PBS), and the cells were subsequently washed with cold PBS before being solubilized in 0.2 N NaOH. The DCF uptake by OATP1B1 was measured using a SpectraMax i3x Multi-Mode Microplate Reader (Molecular Devices, San Jose, CA, USA). To examine the cell surface expression of OATP1B1 and its mutants, we utilized the membrane-impermeable biotinylation reagent NHS-SS-biotin. Briefly, cells in a 12-well plate were labeled with NHS-SS-biotin and then lysed using RIPA lysis buffer. The biotin-labeled proteins were isolated with streptavidin agarose beads, released into Laemmli sample buffer, and separated on a 7.5% SDS-polyacrylamide gel. The transporter protein was detected using the anti-GFP antibody.

### 2.5. Co-Immunoprecipitation

Cells expressing N-OATP1B1-GFP were washed three times with cold PBS and then lysed in immunoprecipitation buffer, which contained 10 mM Tris/HCl (pH 7.5), 150 mM NaCl, 1% NP-40, 2 mM EDTA, 3% glycerol, and protease inhibitors (200 µg/mL phenylmethylsulfonyl fluoride and 3 µg/mL leupeptin). Cell debris was removed by centrifugation at 12,000× *g* for 20 min at 4 °C. The supernatants were transferred to new Eppendorf tubes and pre-cleaned with protein G agarose beads (ThermoFisher). The agarose beads were then removed by centrifugation, and the supernatants were incubated overnight at 4 °C with either anti-GFP or anti-radixin antibodies. Following this, protein G agarose beads were added and mixed with end-over-end rotation at 4 °C. The proteins bound to the agarose beads were then eluted using Laemmli sample buffer containing β-mercaptoethanol and analyzed by immunoblotting with radixin or GFP antibodies.

### 2.6. Site-Directed Mutagenesis

Mutants were generated using a QuickChange Lightning Site-Directed Mutagenesis Kit from Agilent (Santa Clara, CA, USA). The ERM or N-OATP1B1-GFP served as the template for the mutagenesis. All mutant sequences were confirmed through full-length sequencing (ThermoFisher).

### 2.7. Statistical Analysis

Student’s *t*-test was performed when comparing two sets of samples. For comparisons involving more than two groups, the one-way analysis of variance (ANOVA) with Bonferroni’s post hoc test was utilized. Differences between means were considered significant if *p* < 0.05.

## 3. Results

### 3.1. Characterization of N-OATP1B1-GFP Expression and Uptake Function

**Figure 1:**
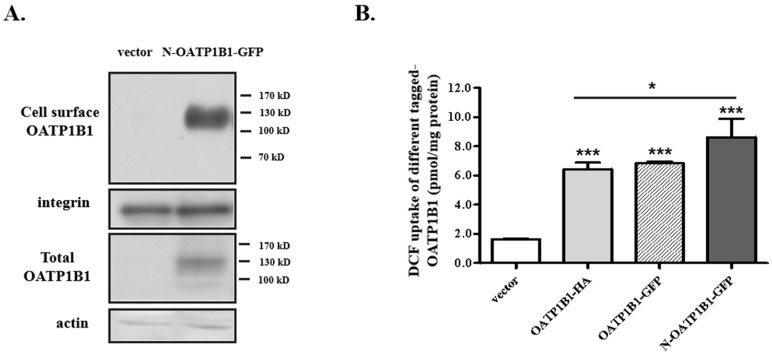
**Characterization of cells overexpressing N-OATP1B1-GFP.** (**A**) Protein expression of cells expressing N-OATP1B1-GFP. Proteins were extracted, labeled with NHS-SS-biotin, pulled down by streptavidin agarose beads, and denatured and separated by SDS-PAGE followed by Western blotting with an anti-GFP antibody. A portion of the isolated proteins (before streptavidin agarose beads pull-down) was directly denatured and used for total protein detection. Integrin and actin were used as the loading control for cell surface and total proteins, respectively. (For the original blots, see Supplementary Figure S1). (**B**) DCF uptake by N-OATP1B1-GFP. Uptake of DCF (1 μM) was measured at 37 °C for 5 min. Net uptake was obtained by subtracting the uptake of cells transfected with the empty vector from cells expressing different tagged OATP1B1. The results represent data from three experiments, with duplicate measurements for each sample. The results shown are mean ± S.D. (*n =* 3). Asterisks indicate significant difference from the vector-transfected control or between different tagged OATP1B1 (* *p* < 0.05, *** *p* < 0.001).

As the carboxyl-(C-) terminus of OATP1B1 may be important for its interaction with regulatory proteins, as demonstrated in the case of its interaction with PDZ, we generated a construct that had a GFP tagged at the amino (N-) terminus of OATP1B1 (designated as N-OATP1B1-GFP) to reduce the interference of the tag on the protein. As shown in Figure 1A, the expression of OATP1B1 was successfully detected with the GFP antibody on the cell surface and in total cell lysates. N-OATP1B1-GFP exhibited a considerable DCF uptake function, which is statistically higher than the cells expressing constructs with the tagging at the C-terminus. The result is consistent with the previous reports showing that the C-terminus of OATP1B1 is important for the function of the transporter [10].

### 3.2. Knockdown of Radixin Increased OATP1B1 Uptake Function

**Figure 2:**
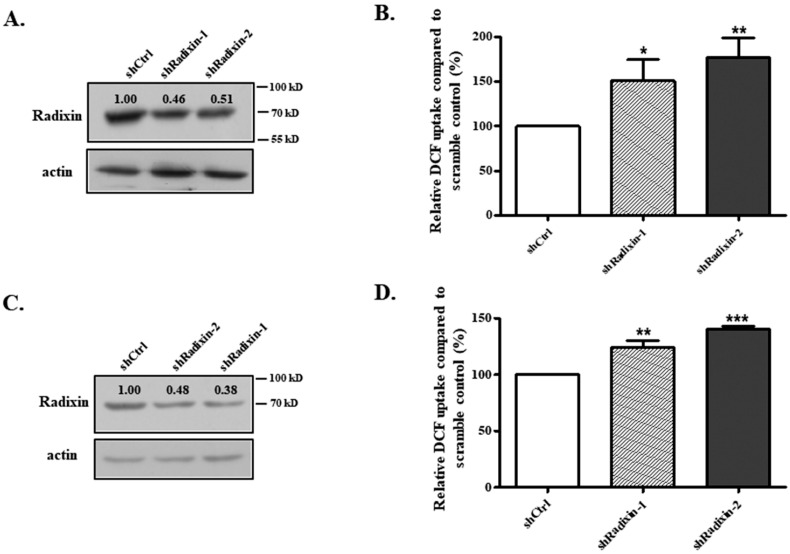
**Knockdown of radixin increased OATP1B1 function.** The protein level of radixin in knockdown HepG2 (**A**) and HEK293 (**C**) cells. Cells were lysed with RIPA buffer, denatured, and then separated by SDS-PAGE which was then followed by Western blotting with an anti-radixin antibody. The same blot was probed with actin as the loading control. (For the original blot see Supplementary Figure S2). DCF uptake function in knockdown HepG2 (**B**) and HEK293 (**D**) cells. The results represent data from three independent experiments, with duplicate measurements for each sample. The results shown are mean ± S.D. (*n* = 3). Asterisks indicate significant difference from scramble control (* *p* < 0.05, ** *p* < 0.01, *** *p* < 0.001).

To investigate whether radixin plays a regulatory role in OATP1B1 function, we generated two radixin knockdown HepG2 cell lines (Figure 2A). When DCF uptake was measured in these knockdown cell lines, the transport activity of OATP1B1 was significantly increased (Figure 2B). To ensure that this response was not specific to a particular cell type, we also developed knockdown cell lines using HEK293 cells (Figure 2C). As demonstrated in Figure 2D, these knockdown cells also showed enhanced DCF uptake, indicating that the effect of radixin abrogation is not cell-type specific. As the response was more robust in the HepG2 cells, we continued the following studies in this liver cancer cell line.

**Figure 3:**
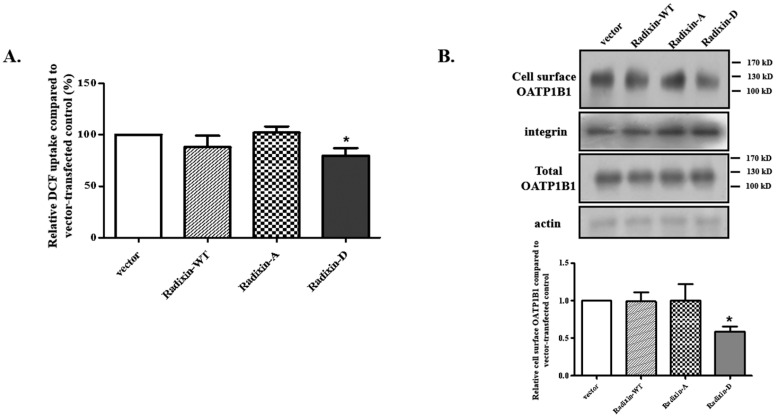
**Effect of radixin overexpression on OATP1B1.** (**A**) DCF uptake by cells overexpressing wild-type and mutants of radixin. The results represent data from three independent experiments, with duplicate measurements for each sample. The results shown are mean ± S.D. (*n* = 3). Asterisks indicate significant difference from vector-transfected control (* *p* < 0.05). (**B**) Cell surface level of OATP1B1 in cells overexpressing wild-types and mutants of radixin. Cells were biotinylated with NHS-SS-biotin and precipitated with streptavidin agarose beads. Proteins were released with Laemmli buffer and then separated by SDS-PAGE, which was followed by Western blotting with anti-GFP antibody. A portion of the isolated proteins (before streptavidin agarose beads pull-down) was directly denatured and used for total protein detection. Integrin and actin were used as the loading control for the cell surface and total proteins, respectively. (For the original blots, see Supplementary Figure S3). A set of representative blots is shown (upper panel). The intensity of the OATP1B1 signal was analyzed by Image J (v1.52) and normalized with integrin. The change was calculated as a fraction of the vector-transfected control, which was normalized to 1. The results represent data from three independent experiments and are shown as mean ± S.D. (*n* = 3). Asterisks indicate significantly different from the control (* *p* < 0.05) (lower panel).

As the reduced level of radixin increased OATP1B1 function, we overexpressed the scaffold protein in HepG2 cells to see whether it suppressed the transporter activity. Unexpectedly, the overexpression of radixin did not affect the activity of OATP1B1. We therefore postulated that it is not radixin per se, but rather its phosphorylation status, which affects the function of OATP1B1, similar to what has been demonstrated in the regulation of MRP2 by ezrin [18]. Radixin is phosphorylated at Thr564, which stabilizes the ERM protein in an open-activated form [19]. Therefore, alanine (phospho-dormant) and aspartate (phospho-mimic) mutants at Thr564 were generated for the investigation. As shown in Figure 3A, radixin-D (T564D) exhibited a significantly suppressed effect on OATP1B1, whilst radixin-A (T564A) showed no effect. Moreover, when the cell surface expression of OATP1B1 was analyzed, it was found that radixin-D significantly reduced the plasma membrane level of OATP1B1 without changing the total protein level of the transporter (Figure 3B). These results suggested that the phosphorylation of radixin is crucial for its regulation of OATP1B1.

### 3.3. Radixin Is Associated with OATP1B1

**Figure 4:**
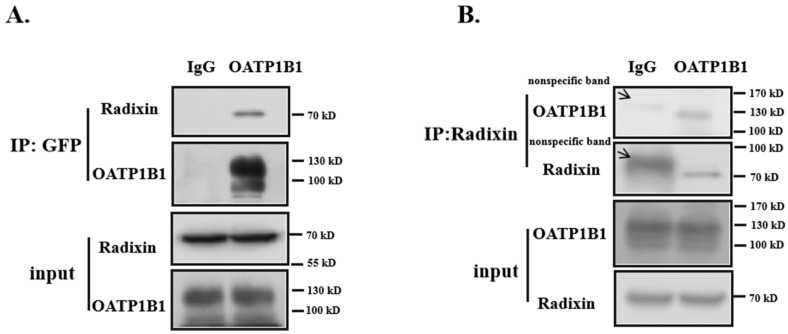
**Interaction of radixin with OATP1B1.** (**A**) OATP1B1-expressing cells were lysed and immunoprecipitated with anti-GFP or normal mouse IgG (as control) and detected with the anti-radixin antibody. (**B**) Cells were immunoprecipitated with anti-radixin and detected with the GFP antibody. (For the original blots, see Supplementary Figure S4).

We next assessed whether the effect of radixin on OATP1B1 occurred through direct interaction. N-OATP1B1-GFP was pulled down by the anti-GFP antibody or normal mouse IgG (as control), followed by immunoblotting with the anti-radixin antibody. As shown in Figure 4A, a signal of radixin was detected in OATP1B1 immunoprecipitates. When proteins from OATP1B1-expressing cells were pulled down by anti-radixin, a specific band detected by the GFP antibody was also observed (Figure 4B), suggesting that OATP1B1 and radixin interact with each other.

### 3.4. PKC-Induced OATP1B1 Internalization Is Likely Mediated by Radixin

**Figure 5:**
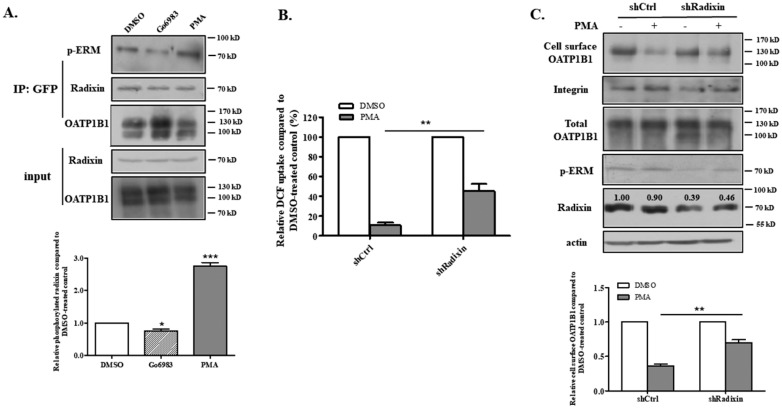
**PKC regulates the phosphorylation status of the radixin that interacts with OATP1B1.** (**A**) The phosphorylation level of the radixin that is associated with OATP1B1. Cells were treated with PMA (10 μM) or Gö6983 (10 μM) for 30 min before cells were lysed, precipitated with the anti-GFP antibody, and detected with phorpho-ERM, radixin, or GFP antibodies. A set of representative blots are shown (upper panel). The intensity of the phosphorylated radixin signal was quantified by Image J and normalized with the total radixin pulled down by the GFP antibody. The change was calculated as a fraction of the DMSO-treated control, which was normalized to 1. The results represent data from three independent experiments and are shown as mean ± S.D. (*n* = 3). Asterisks indicate significant difference from the control (* *p* < 0.05, *** *p* < 0.001) (lower panel). (For the original blots, see Supplementary Figure S5). (**B**) The DCF uptake by cells that had radixin knocked down and treated with PMA. The uptake function was calculated as a percentage of that of the DMSO-treated control, which was normalized to 100% in both shCtrl and shRadixin cells. The results represent data from three biologically independent experiments, with duplicate measurements for each sample. Asterisks indicate significantly different from the control (** *p* < 0.01). (**C**) The cell surface level of OATP1B1 in scramble control or radixin knockdown cells treated with PMA. Cell surface proteins were labeled with NHS-SS-biotin, precipitated, and detected as described above. A set of representative blots are shown (upper panel). (For the original blots, see Supplementary Figure S5). The intensity of the cell surface OATP1B1 signal was analyzed by Image J and normalized with integrin. The change was calculated as a fraction of the DMSO-treated control, which was normalized to 1. The results represent data from three independent experiments and are shown as mean ± S.D. (*n* = 3). Asterisks indicate significant difference from the control (** *p* < 0.01) (lower panel).

The results mentioned above indicate that radixin interacts with OATP1B1, and that the phosphorylation of the scaffold protein may affect the cell surface level of OATP1B1. This, in turn, leads to a decrease in the uptake function of the transporter. The identity of the kinase(s) responsible for phosphorylating the ERM protein remains an area of active research; however, protein kinase C (PKC) is widely regarded as a major kinase involved in phosphorylating ERM, as shown in multiple studies [20,21,22]. Moreover, a previous study from our laboratory demonstrated that activation of PKC increases both the internalization and recycling of OATP1B1, which results in the reduced cell surface expression of OATP1B1 without affecting its total protein level [16]. Therefore, we aimed to investigate whether changes in PKC activity influence the phosphorylation status of radixin. As shown in Figure 5A, activation of PKC with PMA—a potent and selective PKC activator—led to an increase in phosphorylated radixin that was pulled down by OATP1B1. Conversely, inhibiting PKC with Gö6983 resulted in a reduction in phosphorylated radixin co-precipitated with OATP1B1. Additionally, when radixin knockdown cells were treated with PMA, they exhibited a significantly weaker response compared to the scramble control (Figure 5B). Furthermore, while PMA treatment significantly decreased the plasma membrane expression of OATP1B1, it had a much-reduced effect on the cell surface level of OATP1B1 in the knockdown cells (Figure 5C). These findings suggest that PKC may exert its influence on OATP1B1 through the phosphorylation of radixin.

### 3.5. The Role of C-Terminal Positively Charged Amino Acid Cluster for OATP1B1 Interaction with Radixin

**Figure 6:**
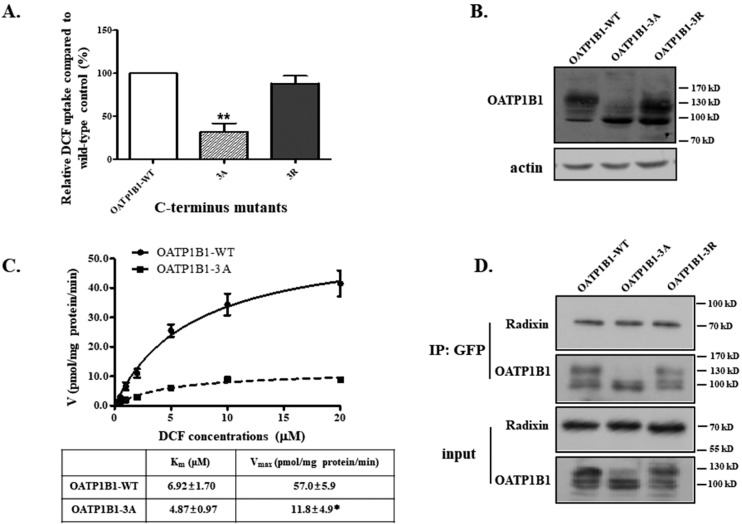
**The carboxyl lysine cluster is important for OATP1B1 function.** (**A**) The DCF uptake by OATP1B1 and lysine cluster mutants. Asterisks indicate significant difference from the wild-type control (** *p* < 0.01). (**B**) The protein expression of OATP1B1 and mutants. (For the original blots, see Supplementary Figure S6). (**C**) The DCF transport kinetics of OATP1B1. Uptake by cells expressing OATP1B1 was measured at concentrations ranging from 0.5 to 20 µM at 37 °C for 2 min. The results represent data from three independent experiments, with duplicate measurements for each sample. The results shown are mean ± S.D. (*n* = 3). K_m_ and V_max_ were calculated using the nonlinear regression of the Michaelis–Menten equation incorporated in GraphPad Prism 8. Asterisks indicate significant difference from the wild-type control (* *p* < 0.05). (**D**) The association of OATP1B1 and radixin in lysine cluster mutants. (For the original blots, see Supplementary Figure S6).

Positively charged clusters at the juxtamembrane position of the membrane protein C-termini are proposed to be a likely motif for the binding of ERM proteins [23]. Upon sequence analysis, it was found that there is a cluster of three lysine residues located at the border of transmembrane helix 12 and the C-terminus (positions 648–650) of OATP1B1. To see whether the motif is important for the binding of radixin with OATP1B1, we generated two triplet mutants, i.e., OATP1B1-3A and OATP1B1-3R, with all three K-mutated to alanine and arginine, respectively. The OATP1B1-3A mutant exhibited a dramatically reduced uptake function, while the R mutant retained comparable activity as the wild-type control (Figure 6A). The reduced function of OATP1B1-3A seemed to be due to the significantly decreased level of the mature form of the protein (Figure 6B), which is the major form that targets the cell surface and is responsible for the transport function of OATP1B1. Kinetic analysis also revealed that the mutant exhibited a much-reduced Vmax, but a minimal change was observed for the Km value (Figure 6C). On the other hand, co-immunoprecipitation demonstrated that neither OATP1B1-3A nor OATP1B1-3R showed an altered interaction with radixin (Figure 6D), suggesting that the triple lysine cluster is not involved in the association between radixin and OATP1B1.

## 4. Discussion

OATP1B1 is an important uptake transporter that plays a significant role in the absorption, distribution, and excretion of various clinical drugs. Alterations in the activity of this transporter protein can affect the pharmacokinetics of therapeutic agents. The current study reveals that OATP1B1 interacts with radixin, a membrane–cytoskeletal crosslinker predominantly expressed in the liver [22]. It was found that the phosphorylation of radixin decreases the cell surface levels of OATP1B1. Additionally, PKC, a kinase previously identified by us that regulates the endocytic process of OATP1B1 [16], is likely involved in controlling the phosphorylation level of radixin and its association with OATP1B1.

Although OATP1B1 has been shown to be regulated by a couple of protein kinases [16,24] and predicted phosphorylation sites have been proposed in high-throughput analysis and prediction servers, the identification of these phosphorylation sites, especially those for the serine/threonine kinases, is still without much success. This promotes one to wonder if the regulation of protein kinases on the transporter is mediated by other proteins. Our current study demonstrated that OATP1B1 was associated with radixin and regulated by the scaffold protein. When radixin was knocked down, the function of OATP1B1 was unexpectedly increased instead of reduced. Moreover, overexpression of the phospho-mimic form of radixin (radixin-D), but not the wild-type or phospho-dormant form of radixin (radixin-A), significantly suppressed the transport activity of OATP1B1, suggesting that it is the phosphorylated form of radixin rather than radixin per se that regulates the function of OATP1B1. When the protein level of OATP1B1 was evaluated, it was found that only the cell surface level of OATP1B1 was reduced, while the total OATP1B1 level was unaltered. Such a phenomenon was similar to our previous finding when studying PKC, which regulated the endocytosis of the transporter through the clathrin-dependent pathway [16]. Therefore, it was postulated that the internalization of OATP1B1 induced by PKC is mediated by radixin. Indeed, the knockdown of radixin was found to significantly attenuate the effect of PMA on the function and cell surface expression of OATP1B1. ERM proteins are scaffold proteins that were demonstrated to be involved in the membrane trafficking of transporters. A previous study of MRP2 revealed that PKC is activated during cholestasis and results in the phosphorylation of ezrin, which causes the internalization and degradation of MRP2 [18]. Interestingly, the activation of radixin, on the other hand, was found to stabilize MRP2 on the plasma membrane [25]. Although ERM proteins are highly homologous to each other and thought to be functionally redundant, studies also point out that each of the members possess overlapping but distinct functions [18]. Radixin is considered the dominant form of ERM protein in primary human hepatocytes, while ezrin was mainly found in immortal human tumor cell lines [26]. Moreover, no interaction between ezrin and OATP1B1 was observed in our current study. Moesin, another member of the ERM proteins, was beyond detectable levels in HepG2 cells, indicating that its absence did not affect OATP1B1 function either. These data suggested that radixin may be the sole ERM protein that regulates OATP1B1 through direct interaction in the hepatocytes.

The activation of PKC was observed in many pathological conditions such as central nervous system dysfunctions, Alzheimer’s disease, neuronal degeneration, and cardiovascular disorders. More importantly, the induction of PKC is closely associated with the promotion and progression of different cancers [27]. Therefore, the activation of PKC in diseases may phosphorylate and activate radixin, which in turn binds to OATP1B1 and reduces its cell surface level. It has been reported that the function and expression of OATP1B1 are suppressed in different liver diseases such as intrahepatic cholestasis, chronic hepatitis C virus infection, hepatocellular carcinoma, non-alcoholic steatohepatitis, and primary biliary cholangitis [28]. However, in most of the above-mentioned cases, the mRNA and protein levels of OATP1B1 are coordinately affected. Whether changes in certain modulators of these pathological conditions will affect the cell surface and/or total protein level is a question worth pursuing in the future.

Our current study using co-immunoprecipitation experiments demonstrated that OATP1B1 and radixin interact with each other. However, there may be other proteins involved in this interaction as well. Therefore, further investigation is necessary to determine whether the relationship between OATP1B1 and radixin is direct or if it is mediated by other proteins. OATP1B1 was recently demonstrated to bind to domain 1 of the scaffold protein PDZK1, which regulates the targeting of the transporter to the plasma membrane [11]. Some PDZ proteins, such as NHERF1 and 2, contain a motif at their C-termini that can interact with ERM proteins and attach the corresponding membrane proteins to the actin cytoskeleton. Although PDZK1 is a PDZ-only PDZ protein, it was found to oligomerize with NHERF1, generating a complicated complex that regulated the targeting, retention, and recycling of various transporters [29]. Since PDZK1 and radixin appear to have opposite effects on the cell surface level of OATP1B1, it is tempting to postulate that these two proteins may work coordinately to control the cell surface level of the transporter. However, this hypothesis needs to be validated through further experiments and more systematic studies.

Although there is no strict consensus site for the interaction of ERM and its associated proteins, it was suggested that a positively charged cluster at the juxtamembrane domain of the protein C-terminus will facilitate the interaction [21]. Although there is such a cluster located at the C-terminus of OATP1B1, the interaction exhibited no change when the three positively charged lysine were mutated to the hydrophobic alanine. Nevertheless, the alanine mutant exhibited significantly reduced uptake function and protein expression, while the mutant that retained the positive charge, i.e., the 3R mutant, showed no change in transport activity and protein level. The results suggested that the positively charged property at these positions may be important for regulating the targeting and/or stability of the transporter protein. A detailed investigation of the roles served by these amino acid residues is currently ongoing in our laboratory.

## 5. Conclusions

In summary, our present study identified radixin as an association partner of OATP1B1. The ERM protein is likely phosphorylated by PKC, and an increase in the radixin phosphorylation level reduces the cell surface expression and function of the transporter protein.

## Data Availability

The authors declare that all the data supporting the findings of this study are available within the paper.

## Supplementary Materials

The following supporting information can be downloaded at: https://www.mdpi.com/article/10.3390/biology14070744/s1, Figure S1: Original blots for Figure 1A; Figure S2: Original blots for Figure 2A,C; Figure S3: Original blots for Figure 3B; Figure S4: Original blots for Figure 4A,B; Figure S5: Original blots for Figure 5A,C; and Figure S6: Original blots for Figure 6B,D.
