# Supplementary material for: The Effect of Radixin on the Function and Expression of Organic Anion Transporting Polypeptide 1B1
Source: Biology (Basel). 2025 Jun 23;14(7):744. doi: 10.3390/biology14070744 (PMC12292255; doi:10.3390/biology14070744)

Supplementary Figure S1 Original blots for Figure 1A

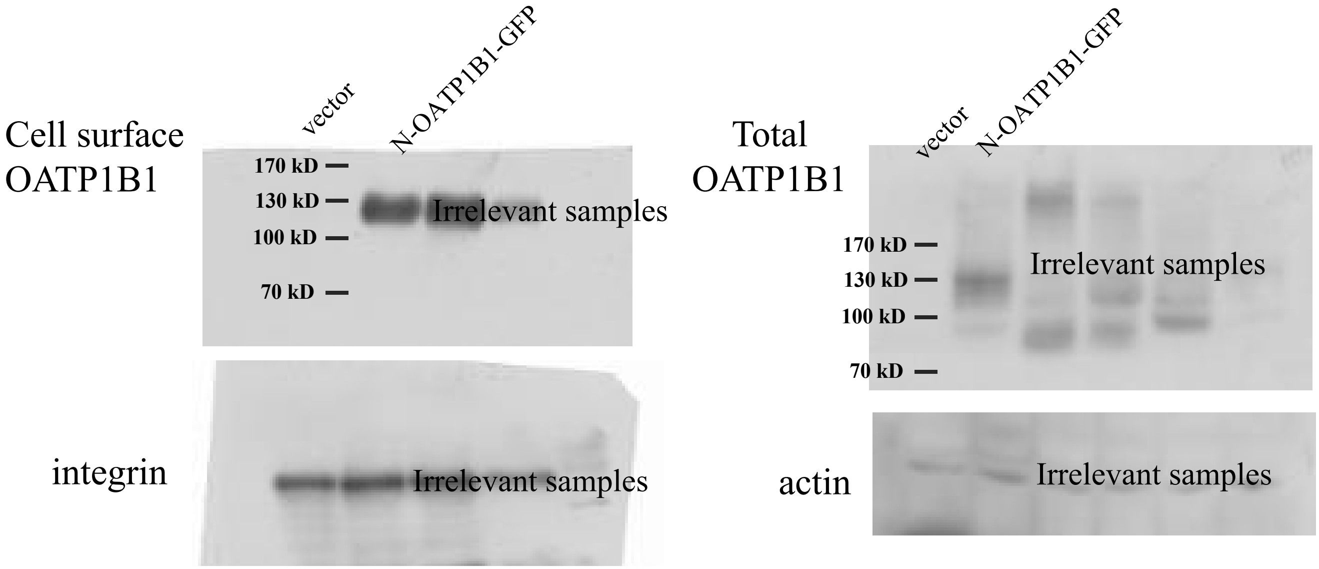

Supplementary Figure S2 Original blots for Figure 2A&C

A.

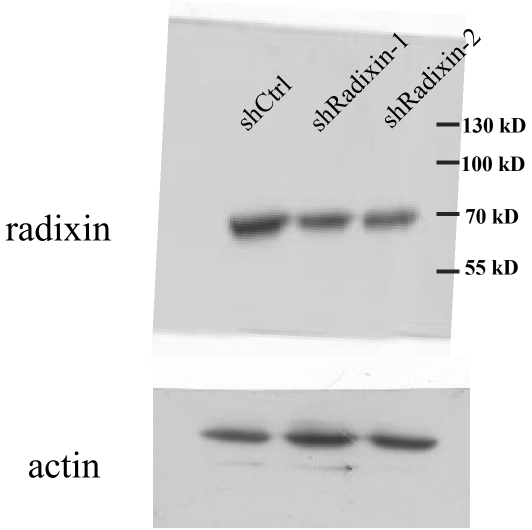

C.

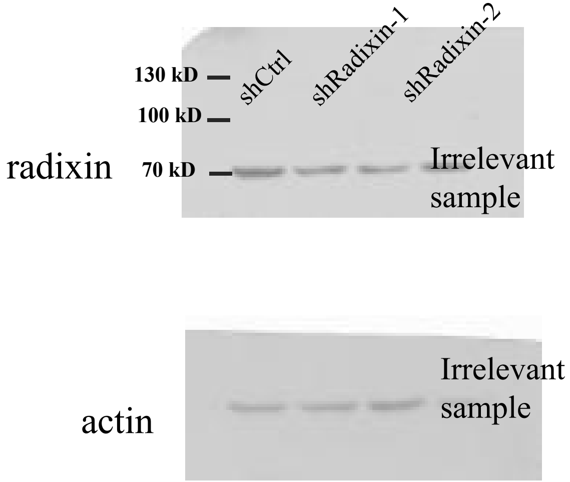

Supplementary Figure S3 Original blots for Figure 3B

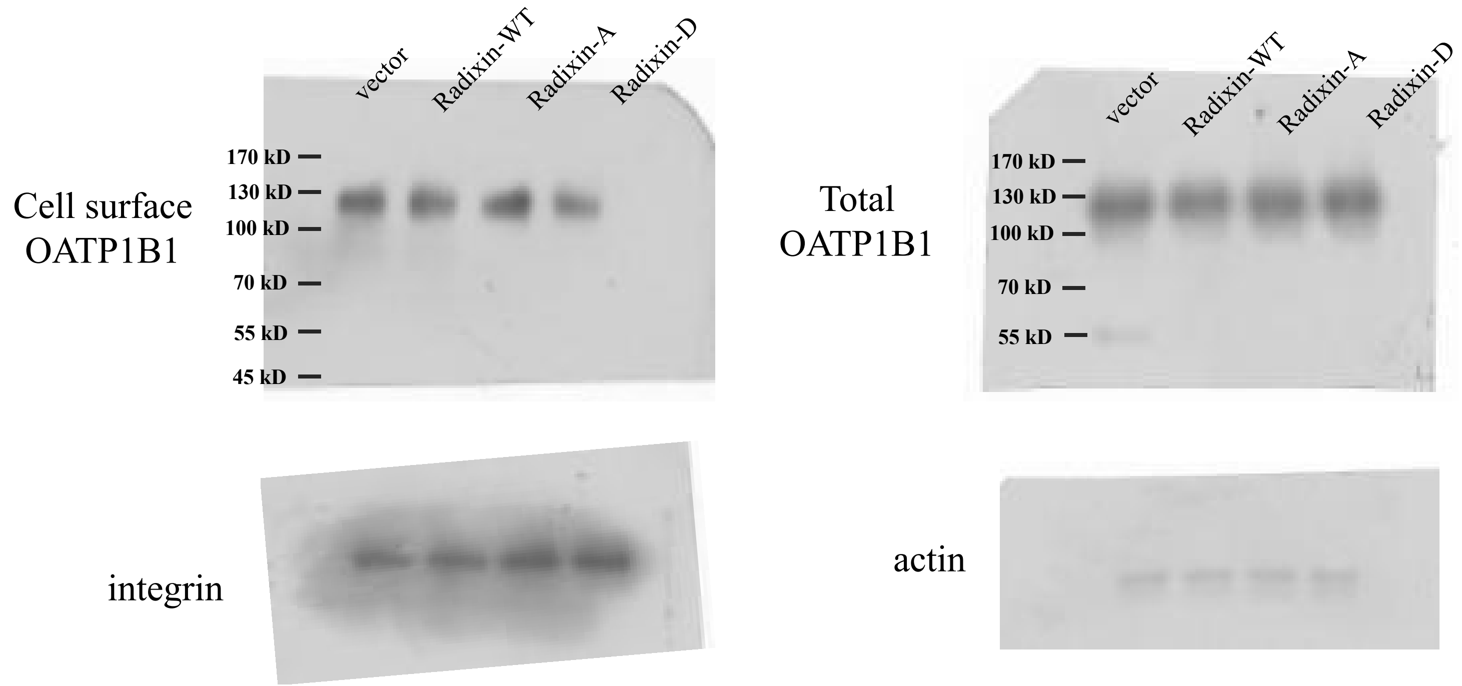

Supplementary Figure S4 Original blots for Figure 4A&B

A.

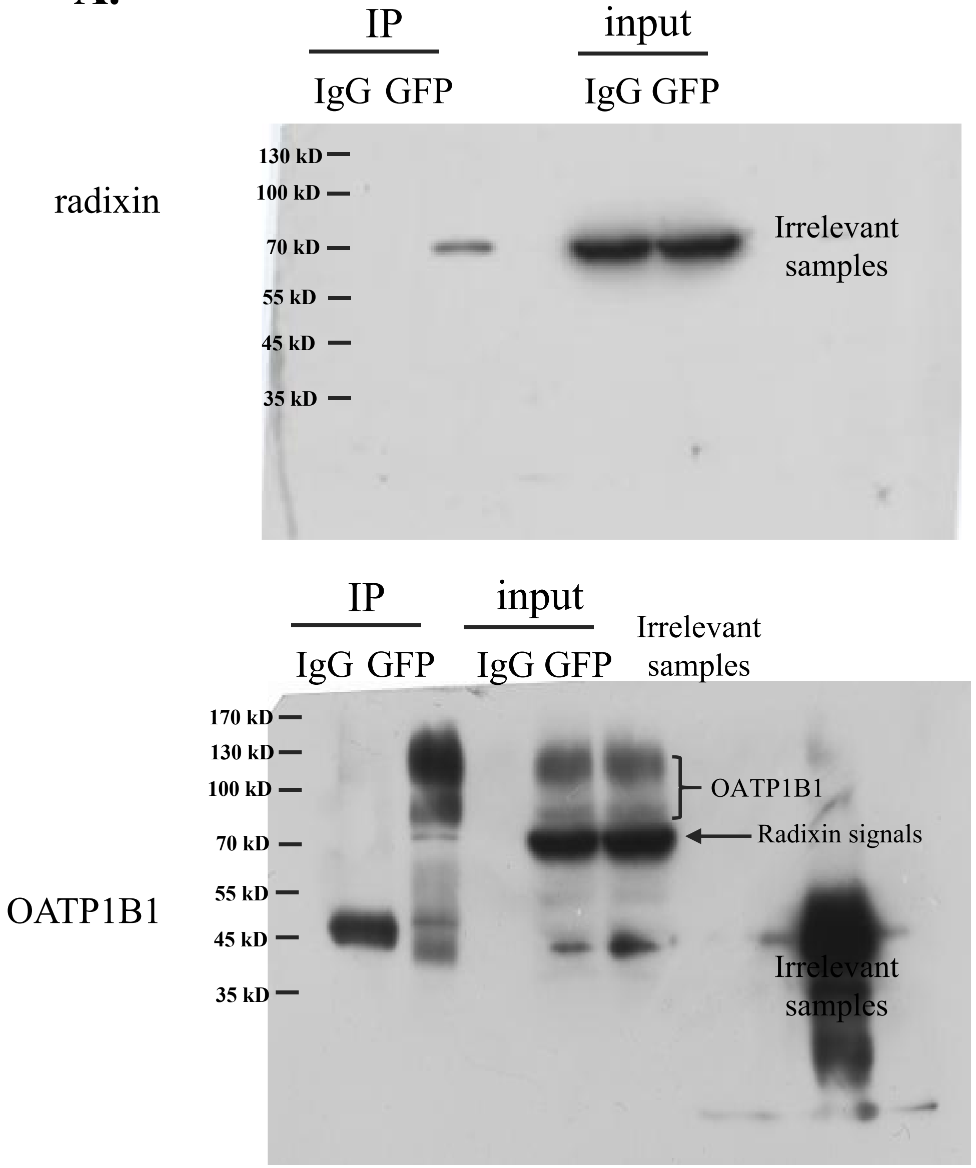

B.

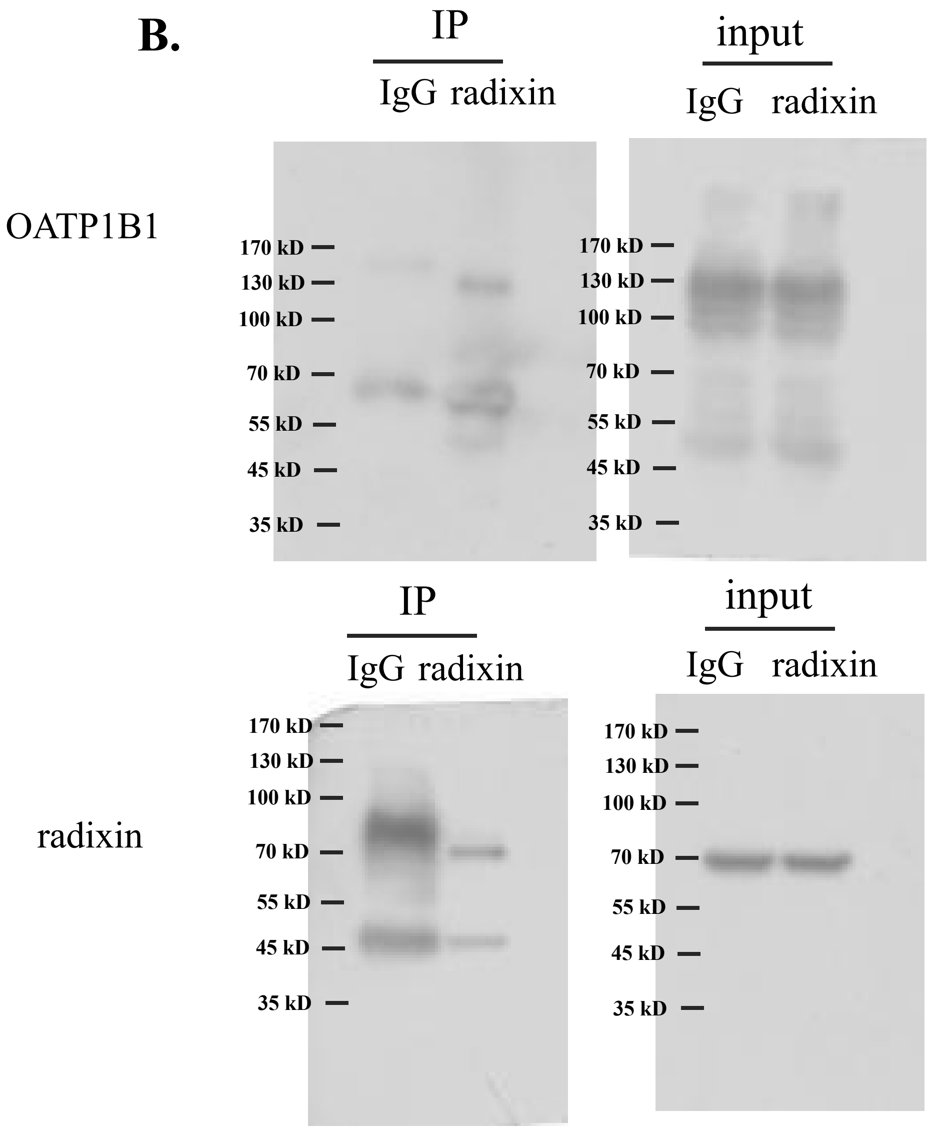

Supplementary Figure S5 Original blots for Figure 5A&C

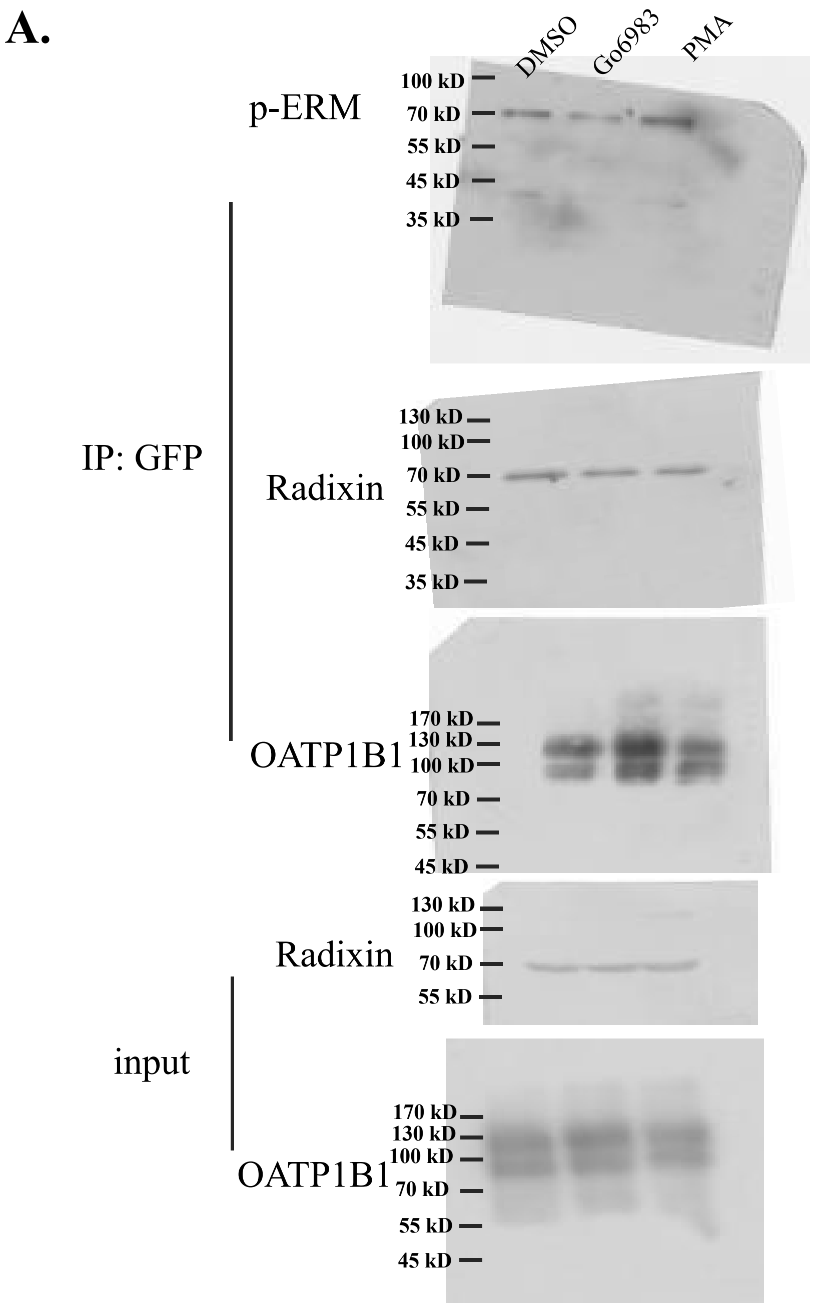

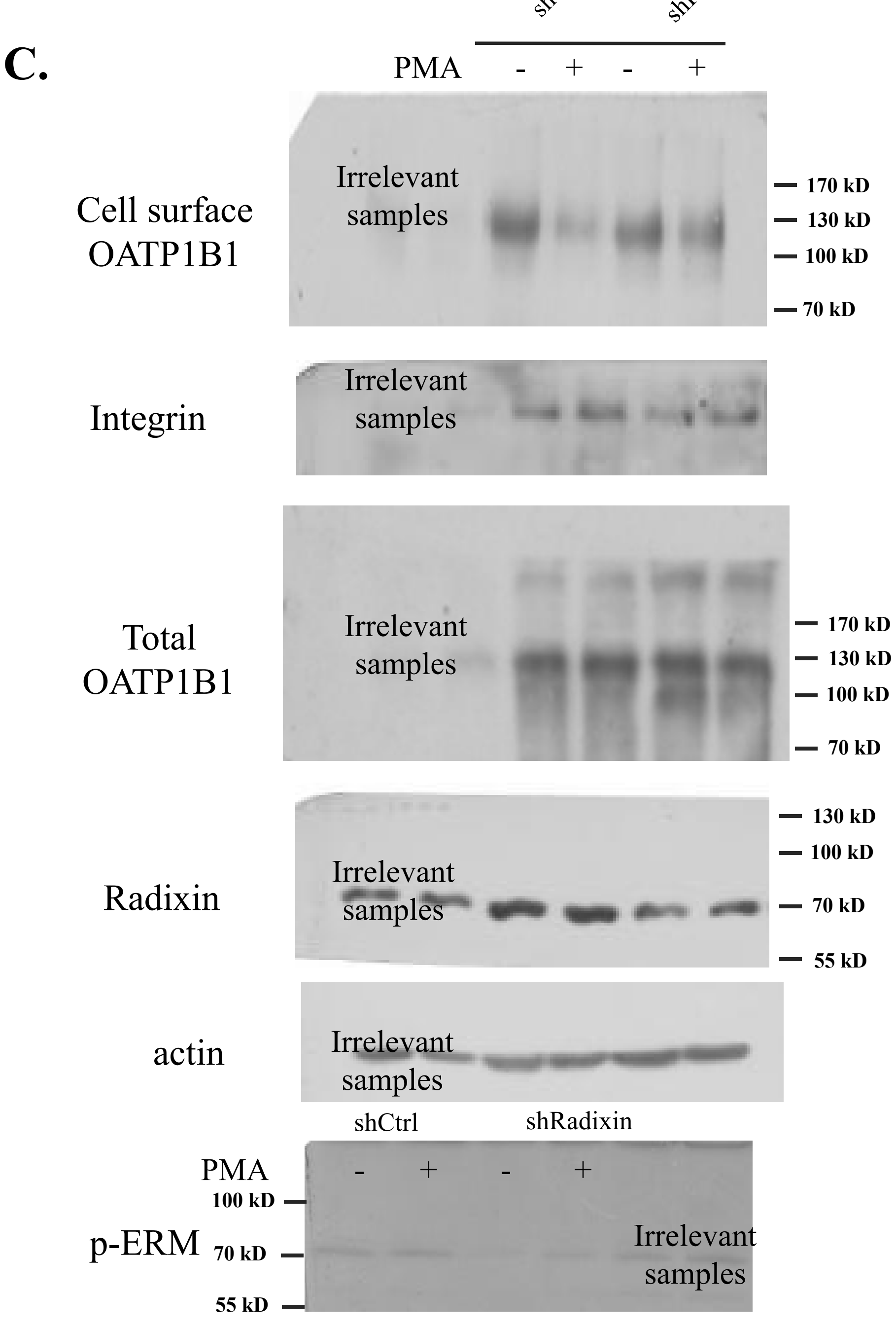

Supplementary Figure S6 Original blots for Figure 6B&D

B.

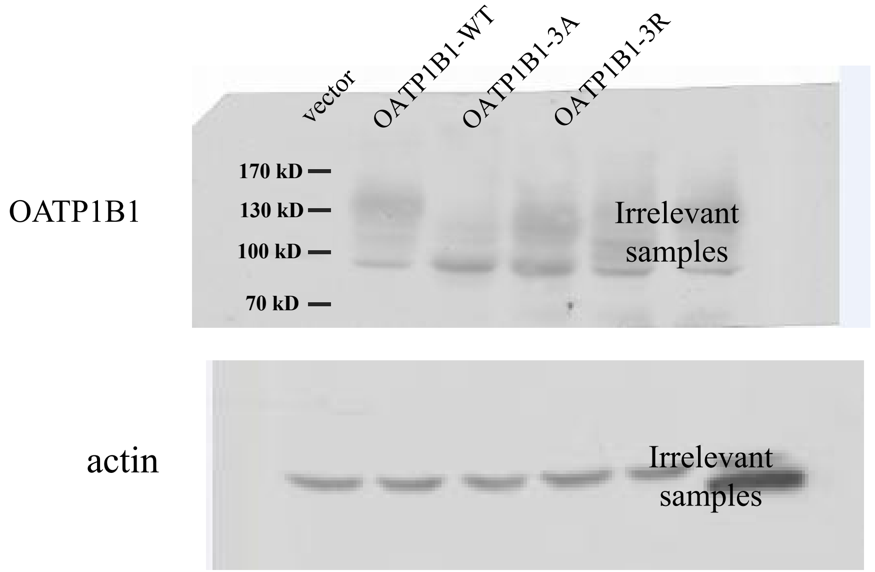

D.

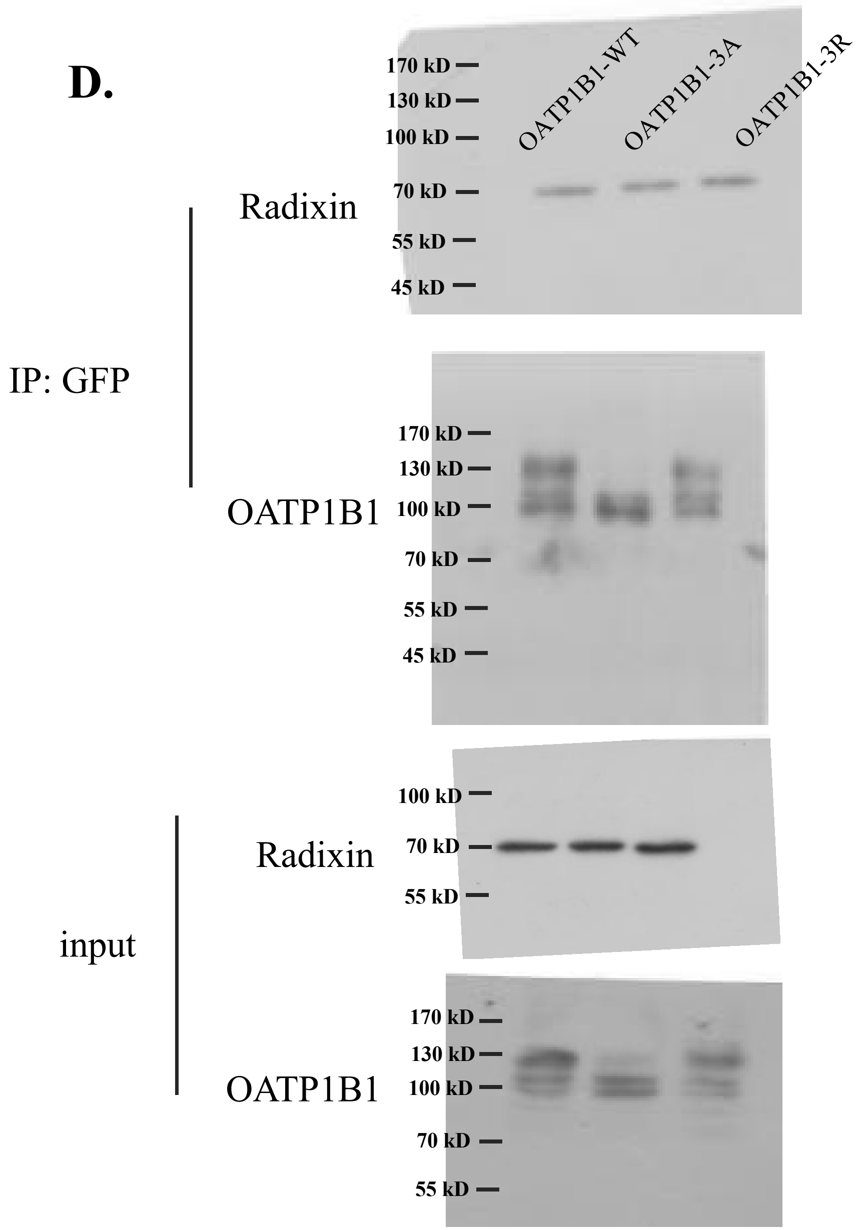

Supplement: Supplementary file 1 [file biology-14-00744-s001.zip › biology-3694683-supplementary.pdf]
